# Design and Implementation Process of an Intelligent Automotive Chassis Domain Controller System Based on AUTOSAR

**DOI:** 10.3390/s25165056

**Published:** 2025-08-14

**Authors:** Yanlin Jin, Yinong Li, Ling Zheng, Guangxuan Li, Xiaoyu Huang

**Affiliations:** 1 College of Mechanical and Vehicle Engineering, Chongqing University, Chongqing 400044, China; 202032021109t@cqu.edu.cn (Y.J.); 202307021325t@stu.cqu.edu.cn (G.L.);; 2 State Key Laboratory of Mechanical Transmission for Advanced Equipment, Chongqing University, Chongqing 400044, China

**Keywords:** AUTOSAR, model-based design, chassis domain controller, software architecture, vehicle software development

## Abstract

With the rapid development of intelligent automobiles, the chassis serves as an essential carrier of intelligence and a necessary condition for achieving high-level autonomous driving. Its electronic and electrical architecture is evolving toward centralized development, which is also significantly increasing the complexity of system functions. Meanwhile, with the integration of more sensors and an increase in data volume, stricter requirements have been placed on software scalability, portability, and maintainability. This paper presents a system software design and implementation approach for the chassis domain controller by integrating the AUTOSAR standard with model-based design (MBD). The developed software is subsequently deployed on a domain controller hardware platform based on the Renesas u2a16 chip for integrated testing. The software algorithm development, model-in-the-loop (MIL) testing, hardware-in-the-loop (HIL) testing, and real vehicle calibration processes are described in detail, focusing on the roll stability control software component in the chassis domain controller. A detailed definition of the toolchain for each development stage is also provided. The feasibility and effectiveness of the proposed chassis domain controller software system development process, based on the combination of the AUTOSAR standard and model-based design, are validated through test results. This method effectively achieves software–hardware decoupling and enhances software scalability, module reusability, and reliability, which is of great significance for improving the efficiency and iteration of chassis domain controller development.

## 1. Introduction

As the automotive industry rapidly advances toward intelligent, electrified, and connected vehicles, digitalization and software-defined automobiles have become the forefront and mainstream of industry development. The automotive industry is transitioning from functional realization to intelligent leadership, with user experience and performance enhancement becoming the focus for major original equipment manufacturers (OEMs) [1,2].

The traditional mode of vehicle controller development involves independent software and hardware development by OEMs and component suppliers without unified standards. This development model results in a strong coupling between software and hardware, with electronic control unit (ECU) software heavily reliant on hardware. When hardware changes occur, they necessitate software redesign, leading to a significant reduction in software portability and maintainability. Moreover, compatibility issues may give rise to potential safety risks. To address this issue, the automotive electronic software development process incorporated the AUTOSAR standard. As an open standardized system architecture for automobiles, it facilitates the independent development of software and hardware through standardized interfaces and a layered design. This effectively resolves issues related to software reuse across different automotive platforms, software safety and reliability, portability, and overall product lifecycle maintainability. It also provides convenience for remote OTA software upgrades in the new generation of intelligent connected vehicles. Furthermore, in response to increasingly stringent vehicle safety requirements and the functional safety standard ISO 26262-1:2018 [3] for road vehicles, AUTOSAR significantly enhances the operational safety of automotive electronic control systems.

As a conduit for the transformation of the automobile from a traditional mode of transportation to an intelligent interactive terminal, the intelligent connected vehicle is witnessing an increasing proliferation of electronic units, leading to a trend of escalating complexity within its systems. Based on distributed electronic–electrical architectures, conventional software development methods exhibit strong coupling between hardware and software. Typically, each function is paired with an ECU, resulting in intricate and inefficient interaction links among ECUs. It is challenging to standardize the development processes and data formats of ECUs across different hardware platforms, significantly diminishing software reusability and portability, thereby impeding the fulfillment of the requirements for the future development of vehicle–road–cloud-integrated intelligent connected vehicles [4]. To address this issue, the next-generation of electronic–electrical architectures for intelligent connected vehicles are rapidly evolving towards domain-centralized and central-computing approaches and are being effectively applied. These architectures involve the upward migration and integrated consolidation of control software for relevant functions into domain controllers for unified management, characterized by more concentrated and efficient computational power, faster communication, customized functionality, and decoupling of software and hardware [5]. The intelligent chassis, serving as an essential prerequisite for achieving high-level autonomous driving and a vital carrier for intelligence, has become a focal point of research and industrial development in recent years. At present, research on chassis area controllers mainly focuses on the coordinated control of chassis domain dynamics. For instance, Yu et al. [6] designed a hierarchical control strategy that takes into account both economy and stability for the coordinated optimization control problem of economy and stability in chassis domain controllers. To enhance the adaptability of the chassis domain controller to various complex working conditions, Shi et al. [7] utilized the Lyapunov exponent to depict the three-dimensional nonlinear stable regions under multiple steering modes, and also investigated the qualitative impact of various driving conditions on vehicle stability. Most existing research focuses on the development of specific algorithms, while there is relatively little research on integrating the development of chassis domain algorithms with the AUTOSAR standard. In light of the rapid development of the intelligent chassis, it is highly necessary to study the development of the next-generation electronic and electrical architecture and the software system of the chassis domain controller [8]. The AUTOSAR architecture offers several benefits, including hardware insensitivity, a layered design that promotes high modularity, and robust security features. As a result, it is an ideal choice for centralized architectures. Integrating the AUTOSAR layered architecture into domain controller software system development presents a new and effective solution to address the increasing demand for intelligent driving capabilities.

AUTOSAR has matured over the years and has become one of the industrial standards for automotive electronic software development [9]. In the development of chassis domain controllers, which require decoupling of software and hardware, as well as high software reusability, AUTOSAR plays a particularly crucial role. AUTOSAR has gradually been applied to automotive electronic control system software development and electronic vehicle design in recent years, yielding significant results [10]. For instance, in vehicle-level communication software design, with the rapid growth of automotive intelligence and autonomous driving, sensors such as lidar, cameras, and millimeter-wave radar have been extensively deployed in the vehicle’s electronic–electrical architecture. This has increased the number of participants in the vehicle network and the processing of critical data, leading to extensive research on data transmission optimization under the AUTOSAR standard [11]. Arestova et al. [12] proposed an integrated solution for modern vehicle communication, combining the AUTOSAR adaptive platform, open platform communications unified architecture, and time-sensitive networking, enabling flexible communication between different devices and reducing the overall cost. Addressing clock synchronization concerns during communication, Luckinger et al. [13] studied precise clock synchronization in CAN networks following the AUTOSAR standard within the automotive hardware and software environment. They reduced clock synchronization jitter through filtering and optimized timestamp processes, achieving a synchronization accuracy of 50 μs. Due to its ability to achieve software–hardware decoupling and define standardized interfaces, AUTOSAR has been widely applied in the development of automotive chassis electronic control system software. Hermans et al. [14] introduced AUTOSAR into the model-based development process to complete the development work for a car’s antilock brake system (ABS) function and carried out HIL verification. Wang et al. [15] described the development process of a pure electric vehicle hierarchical control system software based on the AUTOSAR standard, modularizing vehicle control strategies into different functional modules and designing a runtime environment according to the AUTOSAR standard to ensure data interaction between other available modules and software layers. The developed vehicle controller was tested and validated on a prototype vehicle. Khound et al. [16] developed a central control unit software architecture based on AUTOSAR, demonstrating a complete controller development process based on the AUTOSAR standard and achieving control of a self-balancing robot. In the field of automotive electronic test system development, real-time operating system module testing using AUTOSAR requires a large number of test cases, leading to high overall time consumption. Englisch et al. [17] proposed a method to test AUTOSAR software functionality and timing characteristics by automatically creating test cases in AUTOSAR configuration files to aid in integrating AUTOSAR software into ECUs. Addressing AUTOSAR software-in-the-loop testing, Mihalj et al. [18] proposed an existing advanced driver assistance environment testing system, creating a test environment for communication simulation in the intermediate layer of the AUTOSAR architecture. Furthermore, the AUTOSAR standard has been widely applied in the design of overall vehicle electronic–electrical architectures [19,20], as well as in drive software development [21,22] and other fields. In the research mentioned above, the AUTOSAR standard was introduced to solve the software development tasks of individual functions, achieving software–hardware decoupling and improving software development efficiency.

However, the current development of chassis controllers needs to meet the demands of the electronic and electrical architecture evolving toward domain centralization. Additionally, few studies have applied the AUTOSAR standard to the development of multi-functional software systems such as chassis domain controllers. At the same time, the aforementioned research integrates the AUTOSAR standard with specific functional development, conducts simulation experiments and partial HIL testing, and primarily focuses on the development and implementation of functions, without considering whether the developed software meets the requirements of actual vehicles from the perspective of the overall development process.

In this study, the AUTOSAR 4.2 standard was introduced into the MBD development mode to design the software system architecture of the intelligent chassis domain controller using a hierarchical and modular approach. The designed architecture includes the application layer (ASW), basic software layer (BSW), and real-time environment (RTE). The main contributions of this paper are summarized as follows.

1. Integration of AUTOSAR with MBD to enable a comprehensive, production-oriented development process. We incorporate AUTOSAR 4.2 into the entire V-model development lifecycle of a multi-functional chassis domain controller, spanning requirement definition, system architecture design, application-layer algorithm development, target code generation, MIL, SIL, HIL, and real-vehicle calibration. This end-to-end workflow ensures software–hardware decoupling and facilitates efficient functional iteration, addressing a gap that was not fully covered in previous AUTOSAR-related studies.

2. Multi-functional software architecture covering core chassis control modules. In contrast to previous approaches that focus on individual functions, such as RSC, our application layer integrates multiple functionalities—including ABS, ASR, AYC, BM, and a newly developed RSC module—within a unified, AUTOSAR-compliant architecture. This design enables seamless functional coordination through an arbitration software component (SWC) and enhances modular reusability across different platforms.

3. Validation across MIL, HIL, and full-scale vehicle tests. Few studies have demonstrated the development of AUTOSAR-based chassis domain controllers that were validated through actual vehicle testing under extreme, rollover-prone conditions.

The rest of this paper is organized as follows: Section 2 introduces the software system design of the chassis domain controller based on AUTOSAR. Section 3 presents the design and engineering implementation process of the application layer software components. Section 4 describes the software-in-the-loop (SIL) test, HIL test, and real vehicle test processes. Section 5 concludes the paper.

## 2. Design of Chassis Domain Controller Software System Based on AUTOSAR

As the functionalities of the automotive chassis and the driving tasks it supports continue to expand, the complexity of the chassis domain control system is constantly increasing. Each stage of the development process adds sophistication, and any hidden issues during development could cause significant damage to the entire system. Numerous autonomous driving companies and original equipment manufacturers are gradually adopting model-based design methods in system software design to enhance code maintainability and comprehensibility. This approach enables comprehensive, multi-round testing of each module throughout the entire design process, thereby avoiding frequent modifications of algorithm logic during on-site debugging and improving the efficiency and design proficiency of the controller.

### 2.1. Model Design Concept

In the traditional software development process, tasks such as requirements analysis, design, implementation, and testing are often executed sequentially on different development platforms. The defects that exist in the previous phases are accumulated layer by layer, leading to a significant consumption of time and resources during the testing phase. In contrast, the MBD approach uses models to clarify development requirements and specifications, ensuring that design and analysis are synchronized. This method can capture and remove errors before system-level integration, and automatically generates production-level code and test cases from the model. Finally, software testing is completed based on requirements. The MBD approach improves overall development efficiency and reduces the total development cost.

**Figure 1:**
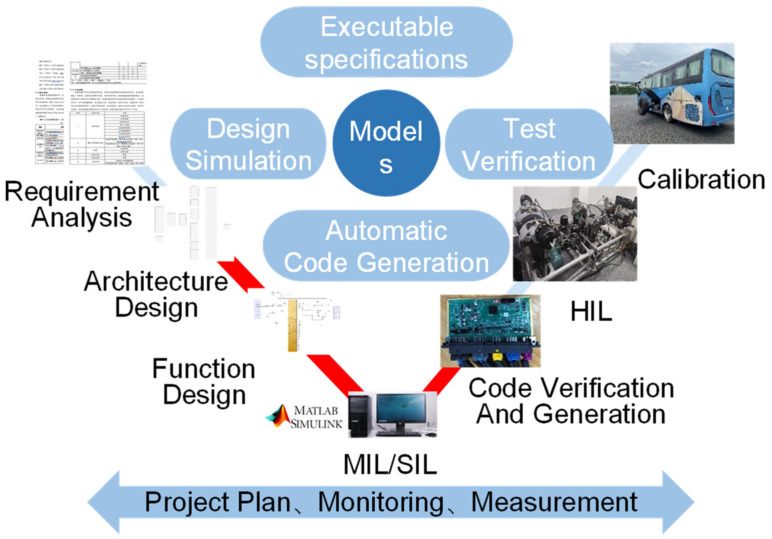
Chassis domain controller V-models development process.

For the development of the automotive chassis domain controller, this article introduces the most commonly used V-model development process in MBD. The AUTOSAR standard is integrated into the software system’s V-model development process, with the development lifecycle of the V-model shown in Figure 1. The functional development process follows the top-down linear process on the left side of the V, while verification and testing are carried out in a bottom-up manner on the right side of the V. The system can use the same development, compilation, and testing environments, and each process can be thoroughly validated. The AUTOSAR standard primarily focuses on software architecture, module design, and implementation, as indicated by the red blocks within the V-model development process.

The development process of the chassis domain controller is based on the V-model development approach. It encompasses requirement definition, system architecture design, functional development, target code generation, HIL simulation testing, vehicle-level testing, and calibration. The system architecture design, functional development, and target code generation are carried out in compliance with the AUTOSAR standard, encompassing the following specific content.

1. Requirement Definition: The specific requirements and performance standards for the software functional components that need to be implemented—such as ABS, ASR, ESC, BM, and state parameter estimation—are analyzed based on the specific functions of the chassis in the longitudinal direction. The hierarchical relationships among the functions within the software architecture are analyzed, and a user requirement specification document is generated. This document encompasses system functionality, performance, data, and security.
2. System Architecture Design: The software components (SWCs) are categorized according to their intended functions, followed by the analysis and determination of the data types and communication interfaces for each SWC. A communication matrix is utilized to identify the communication ports for each SWC in the CAN network, and a communication mapping table is generated to map the signals to the ECU’s external ports. Subsequently, the Software Architecture module in the AUTOSAR Blockset of Matlab/Simulink is employed to manage the system’s architecture and develop the model framework based on the function’s dependency relationships. Upon completion of the design, the ARXML file containing information such as Runnable, Interface, Datatype, and more is automatically generated. This step also involves performing low-level software development BSW work, including configuring the OS module for task scheduling, the EcuM module for ECU management, the BswM module for BSW management, and the communication modules. The relevant configuration work is carried out using the Vector-AUTOSAR toolchain.
3. Function Development: The control algorithms for various functional modules are built based on the system architecture using the MATLAB/Simulink modeling tool, and MIL testing is conducted to verify and promptly correct any functional errors in the models. To facilitate subsequent HIL testing and vehicle calibration, the model must be equipped with the necessary calibration and observation parameters in this environment.
4. Target Code Generation: The model is transformed into product code that complies with the AUTOSAR standard by mapping individual elements of the code based on the AUTOSAR Component module and using Embedded Coder. Finally, an integrated compilation of all programs is performed in the Green Hills compiler to generate the executable file.
5. HIL Testing: The generated code is downloaded into the chassis domain controller with the Renesas u2a16 MCU. The test object is connected to the real-time processor through the I/O interface, and the simulation model runs on the host computer to observe and record the operational status of the controlled object.
6. Testing and Calibration: The domain controller is mounted on the actual vehicle. At the experimental testing base, the functions and performance of the domain controller are calibrated by adjusting the model’s calibration parameters. For the chassis domain controller, it is necessary to complete high-temperature, high-altitude, and low-temperature testing and calibration work.

**Figure 2:**
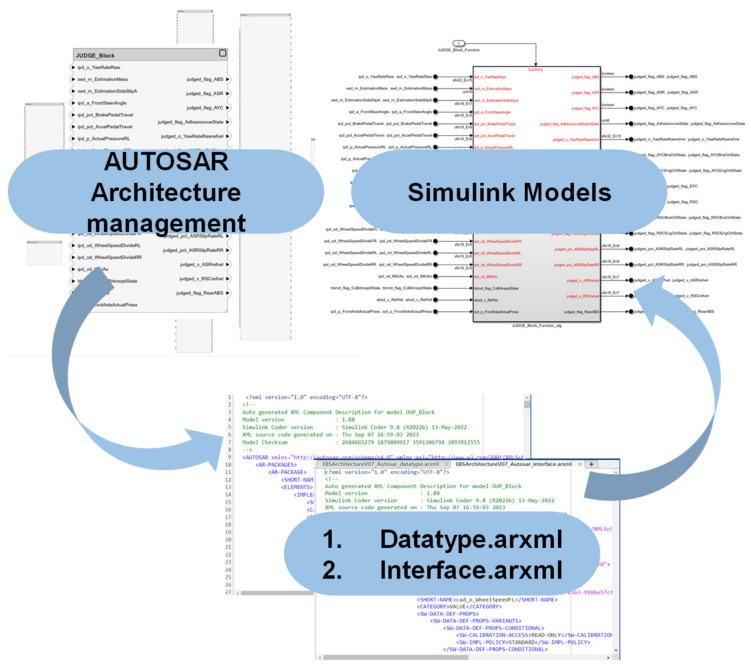
Top-down development process.

**Figure 3:**
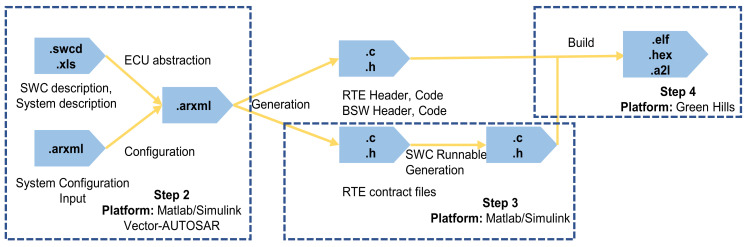
AUTOSAR architecture phase output.

The AUTOSAR standard follows a top-down process overall, primarily applying steps 2 to 4. The process is shown in Figure 2, and its outputs at each stage are shown in Figure 3.

### 2.2. Chassis Domain Controller Software Architecture Design

The design of the software architecture is crucial for realizing the functions and meeting the requirements of the chassis domain controller. This section elaborates on the core architecture based on the AUTOSAR standard. Adhering to AUTOSAR principles ensures modularity, scalability, hardware independence, and facilitates integration and maintenance. We first introduce the basic concepts of the AUTOSAR layered architecture and then detail the specific design of each layer in the chassis domain controller environment, namely, the ASW, the RTE, and the BSW.

#### 2.2.1. Introduction to the AUTOSAR Architecture

The aim of the AUTOSAR standard is to establish a standardized automotive software architecture among automotive OEMs and suppliers. This standard is intended to address the increasingly complex challenges in automotive software and E/E architecture against the backdrop of expanding automotive functionalities by decoupling automotive application software, essential software, and hardware. Its objective is to enhance cross-platform compatibility, efficient reusability, rapid updating, and maintenance capabilities of the software, thereby reducing the development cycle and costs associated with automotive software.

**Figure 4:**
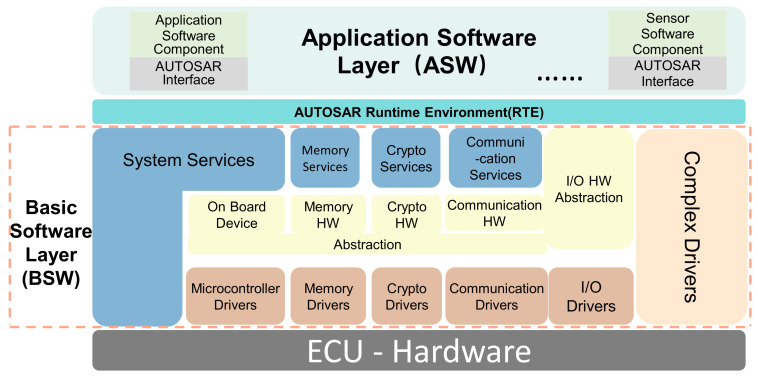
AUTOSAR’s layered software architecture [23].

The AUTOSAR standard architecture is shown in Figure 4 [23]; the whole architecture, from top to bottom, can be divided into three layers: ASW, RTE, and BSW. Each layer is modularized through micro-SWCs, and interactions between modules are realized via standardized interfaces to ensure module independence.

The ASW comprises several SWCs, each of which may contain one or multiple runnable entities (REs). The RTE layer is an intermediary component for managing communication between SWCs and the BSW layer. Different SWCs can interact through a virtual functional bus (VFB), which abstracts the communication between SWCs, the ASW, and the BSW, providing a foundation for SWCs independent of hardware [24]. This enables SWCs to communicate within the same ECU and across multiple ECUs using the VFB, with two allowed modes: send–receive interfaces (S/R) communication, where one SWC sends information and another SWC receives it, and client–server (C/S) communication, where the client requests a service and the server provides it. The BSW provides system services and hardware drivers for the ASW layer and SWCs to execute specified tasks. BSW mainly consists of the service, ECU abstraction, and microcontroller abstraction layers (MCAL). By abstracting the ECU, BSW enables the replacement of ECUs without requiring modifications to the upper-layer applications, achieving software and hardware-decoupled development. In the chassis domain control software systems, SWCs such as ABS, ASR, and ESC are deployed in the ASW layer. In contrast, the BSW layer provides the foundational software environment and services for application functionality. The ASW layer communicates with the BSW layer and other ECUs through standardized interfaces provided by the RTE layer.

#### 2.2.2. Chassis Domain Controller Application Layer Design

**Figure 5:**
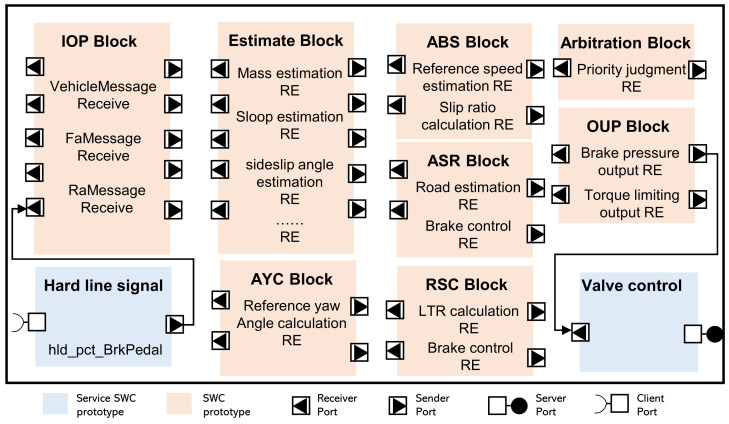
Application software layer component structure block.

The ASW of the chassis domain controller is primarily designed to achieve fundamental braking and to utilize braking to generate additional lateral force and torque to maintain vehicle stability. Based on the similarity of use scenarios and the convergence of the required information to integrate the functions, the application layer of the chassis domain controller can be divided into input processing SWC, state estimation SWC, longitudinal function SWC, arbitration SWC, and output processing SWC. The application software architecture and data flow of the chassis domain controller are illustrated in Figure 5. Partitioning the application layer into several functionally similar modular components allows for the abstraction of data flow and service invocation relationships between the modules, reducing coupling and facilitating precise fault and issue localization. This approach benefits system software updates and upgrades.

Several runnable entities are contained within each SWC. These entities represent the specific implementation of functions and record information such as trigger conditions and interface lists. The input processing SWC obtains input data from the RTE layer and performs signal filtering, vehicle speed calculation, and unit conversion within the module. The state estimation SWC is responsible for estimating the vehicle’s mass, center of gravity, lateral inclination angle, road slope, and road adhesion coefficient. The parameters obtained can be used in subsequent longitudinal function modules to achieve good braking performance and vehicle stability under different loading and operating conditions. The longitudinal function SWC mainly includes the BM, ABS, ASR, AYC, and RSC modules, which generate additional lateral force torque through braking to maintain vehicle stability. The four-wheel braking pressure and engine torque limit signals are outputs of the longitudinal function modules. Under the same operating conditions, multiple longitudinal function triggers may occur, and the arbitration SWC is used to coordinate the signals and output a unique braking control signal. The output processing SWC performs unit conversion on the output signals and sends data to the RTE layer to control the hardware.

#### 2.2.3. Runtime Environment Layer Design

The RTE, positioned between ASW and BSW, represents a tangible implementation of the AUTOSAR VFB. It aims to facilitate data transfer between SWCs and BSW by establishing relevant interface functions. The RTE layer features two primary types of interfaces: unidirectional S/R and bidirectional C/S interfaces. This study uses the unidirectional S/R interface for the input signals and output commands between SWCs. In contrast, the bidirectional C/S interface is employed for the interaction between SWCs and the underlying layer, such as the brake pedal opening signal and the solenoid valve opening/closing signal.

#### 2.2.4. Basic Software Layer Design

**Figure 6:**
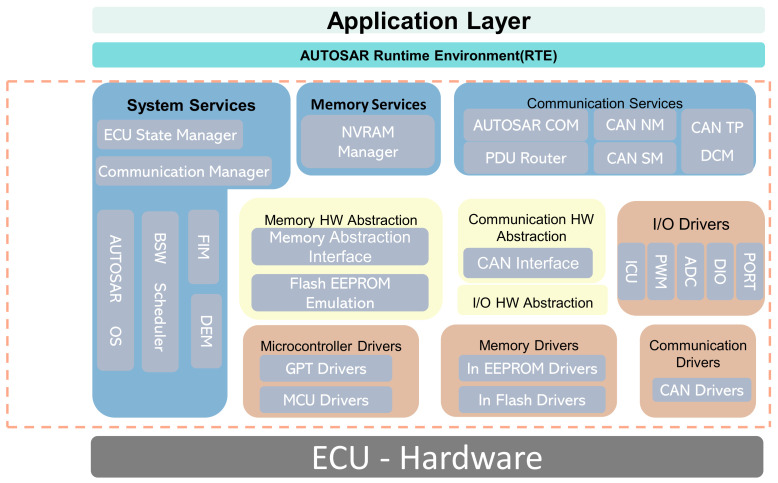
Basic software layer structure block.

The BSW provides essential infrastructure services such as peripheral drivers, memory management, task scheduling, and communication for the specified tasks required by the ASW, acting as a bridge between the application software and the microcontroller. The BSW layer structure designed in this paper, as shown in Figure 6, includes the Service Layer, ECU Abstraction Layer, and Microcontroller Abstraction Layer. The driver code is deployed in the MCAL, which isolates the upper-layer software from hardware resources. Access to hardware is achieved through MCAL device drivers. The hardware drivers designed in this paper are mainly categorized into Microcontroller Drivers, Memory Drivers, Communication Drivers, and I/O Drivers, thus providing an application programming interface for hardware devices and microcontrollers. The ECU Abstraction Layer abstracts and encapsulates the entire ECU, providing a unified interface for accessing storage, communication, and I/O, and facilitating interaction between the Service Layer and MCAL. The Service Layer offers communication, storage, and system services.

The software architecture of the chassis domain controller system designed in this paper is based on the AUTOSAR specification. In this architecture, the software is developed at the ASW layer using the AUTOSAR Blockset in the Matlab/Simulink environment, with the Software Architecture module serving as an architectural management tool. SWCs are constructed and subjected to relevant simulation testing in Simulink. After passing AUTOSAR code specification checks in the AUTOSAR Component module, the model generates production code using Embedded Coder. The BSW is configured using the Vector-AUTOSAR toolchain. The specific design and implementation of the chassis domain controller software based on AUTOSAR is accomplished using the methods described above. Finally, the Green Hills compiler integrates and compiles all programs into executable files.

This paper’s design follows the ISO 26262:2018 framework for functional safety in road vehicles, focusing on hazard analysis, safety goal derivation, and safety mechanism implementation at the software level. The AUTOSAR layered architecture facilitates safety compliance by isolating application software from hardware, enforcing standardized interfaces, and supporting redundancy in communication and control pathways.

In the event of sensor failure or data corruption, the affected functional module (e.g., RSC) transitions to a safe fallback mode using estimated parameters from the State Estimation SWC. If critical safety-related data (e.g., steering angle) is lost, the arbitration SWC inhibits certain stability interventions and reverts to base braking functionality to preserve vehicle controllability.

In the actual development process, MIL fault injection was carried out. During the MIL testing period, simulated sensor signal loss, signal noise spikes, and data transmission delays were introduced to confirm the fault detection and safety state behavior. HIL robustness testing was conducted to simulate communication errors, controller area network (CAN) message delays, and ECU overload scenarios to verify the recovery program’s capabilities. For vehicle-level safety verification—during the actual vehicle testing period—non-critical sensor disconnection operations were performed under controlled conditions to observe the system’s backup mode and ensure that no unsafe intervention occurred.

## 3. Application Layer Software Component Design and Engineering Implementation

The chassis domain controller application layer is composed of the input processing SWC, state estimation SWC, longitudinal function SWC, arbitration SWC, and output processing SWC. The architectural design and configuration of the corresponding SWCs are completed in the Software Architecture, with the respective ARXML files being exported. Module-based graphical development and code generation are carried out based on the architecture in MATLAB/Simulink. The longitudinal function SWC of the chassis domain controller designed in this paper contains multiple operational entities, each employing different algorithms to achieve predefined functions. To provide a detailed illustration of the software development process of the chassis domain controller, this paper offers an in-depth description of the design and implementation process of the RSC component.

**Figure 7:**
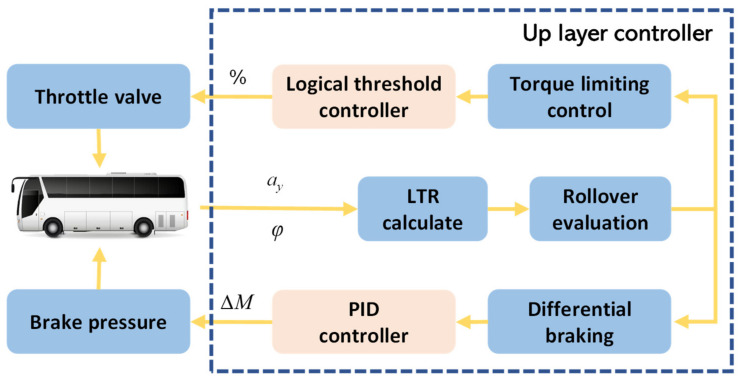
RSC control logic. (*a_y_* represents the lateral acceleration, *φ* represents the roll angle of vehicles, ∆*M* is the additional tilting moment).

Large buses are more prone to rollover accidents than other vehicles when turning due to their heavy load and high center of gravity. The RSC function assesses the rollover risk and applies an additional lateral tilt control torque to the entire vehicle in advance through differential braking, effectively preventing rollover accidents. This paper focuses on large buses and employs a combined limited engine torque and differential braking control strategy to achieve rollover prevention. First, the rollover risk is estimated based on the lateral load transfer ratio (LTR), which categorizes the danger level into mild and severe. For mild danger, only limited torque control is triggered, using the PID control method to calculate the engine’s limited torque value and sending a limited torque percentage command to the engine ECU to reduce the engine torque output and thus decrease the vehicle speed. For severe rollover danger, both limited torque control and differential braking are triggered, using the PID control method to calculate the additional tilt control torque and apply differential braking to the vehicle based on the tilt control torque distribution scheme. Simultaneously, a limited torque percentage command is sent to the engine ECU to reduce the vehicle’s rollover risk. The specific control logic diagram is shown in Figure 7. In contrast to the traditional rollover prevention control, which only utilizes differential braking, this paper fully considers the role of engine torque limitation in reducing speed. It combines limited torque control with differential braking, categorizes rollover risk, and enhances rollover prevention while also improving ride comfort.

The upper-level controller is mandated to estimate the rollover risk status of the vehicle and generate the corresponding additional roll control moment. In real time, the rollover risk estimation module operates to assess the likelihood and magnitude of rollover risk. Various control methods are employed to mitigate the risk of rollover. Specifically, this paper utilizes the lateral load transfer ratio method for rollover risk estimation, which determines the rollover risk by detecting whether one side of the vehicle’s wheels is lifted off the ground [25]. Roll control aims to ensure that the vehicle operates below the LTR threshold, which defines the ratio of the difference in vertical load between the wheels on the left and right sides to the vehicle’s total weight. The expression for LTR is as follows:

(1) $$LTR=\frac{F_{L}-F_{R}}{mg},$$

where *F_L_* represents the vertical load on the left wheel, *F_R_* represents the vertical load on the right wheel, *m* represents the vehicle mass.

From the cost and practical point of view, the vehicle will not be equipped with wheel vertical load sensors. However, the LTR can be estimated based on the state of the vehicle and the force balance relationship, taking the center of lateral tilt as the origin, while ignoring the rotation effect when the vehicle is tilted sideways. The moment balance relationship of the body around the x-axis is the following:

(2) $$F_{L}\frac{L}{2}+m_{s}a_{y}h_{s}\cos\varphi +m_{s}gh_{s}\sin\varphi =F_{R}\frac{L}{2},$$

where $m_{s}$ is the mass of the spring load, $h_{s}$ is the distance from the center of mass to the center of lateral inclination, *φ* is the roll angle of vehicles, $a_{y}$ is the lateral acceleration, and *L* is the wheelbase.

The unsprung mass of the bus studied in this paper accounts for a small proportion of the total vehicle mass, while the vehicle’s lateral inclination angle is generally very small. Based on the assumption of small angles, the following relationship can be obtained:

(3) $$\left\{\begin{array}{c} \cos\varphi =1\\ \sin\varphi =\varphi \\ m_{s}=m \end{array}\right.,$$

Joining Equations (1)–(3), the expression for LTR is obtained as follows:

(4) $$LTR=\left|\frac{2a_{y}h_{s}-2g\varphi h_{s}}{gL}\right|,$$

The paper divides the LTR into gradients, considering LTR ≥ 0.8 as having a significant risk of rollover, 0.65 < LTR < 0.8 as having a slight risk of rollover, and 0 < LTR < 0.65 as having no risk of rollover. When there is a slight risk of rollover, direct engine torque intervention is applied. When there is a significant risk of rollover, a PID controller is used to calculate the required additional roll moment based on the actual LTR and the threshold of 0.8. Furthermore, the additional roll moment is converted into the controlled strategy’s braking moment, as shown in Equation (5):

(5) $$T=\frac{2\Delta MR}{L},$$

where ∆*M* is the additional tilting moment, *T* is the braking torque, and *R* is the wheel radius.

The braking torque–braking pressure curve obtained from actual vehicle testing is used to derive the braking pressure as the actual control signal output. The allocation of braking pressure is further determined, with the allocation program detailed in Table 1. The outer wheel is accurately determined based on the steering wheel angle *δ*, which is measured by the steering wheel sensor. By applying the brakes to the vehicle’s outer wheels, the vehicle’s sway angular velocity can be effectively reduced, and the corresponding lateral acceleration can also be suppressed. This indicates that braking the outer wheels is more effective in reducing the LTR.

## 4. Performance Evaluation and Results

To verify the software functions and performance of the chassis domain controller developed based on AUTOSAR, an ECU based on the Renesas U2A16 chip was adopted as the hardware platform. Combined with the calibration test on the real vehicle, the MIL test was carried out on the software side, and the HIL test was conducted on the hardware side. The real vehicle test verification was also completed at the national standard test field. The vehicle parameters used are shown in Table 2.

### 4.1. Model-in-the-Loop Test

**Figure 8:**
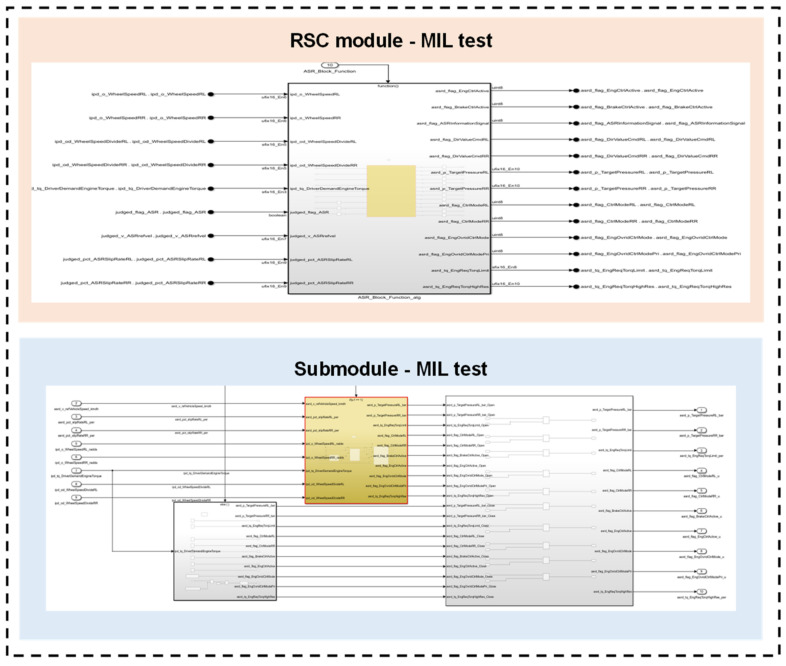
RSC module MIL testing.

The chassis domain controller designed in this paper was developed in accordance with the MBD process. This involves the creation of test cases encompassing inputs and outputs for conducting MIL testing on individual and integrated modules during the application software design phase. The testing results are subsequently compared to validate whether the control algorithm model meets the design requirements. MIL testing identifies and rectifies software errors and logical flaws early on, thereby significantly reducing development time and conserving development costs. The application layer software MIL testing for the chassis domain controller was carried out in Matlab/Simulink, with test cases written for a 2065 unit module and integrated module. The constructed test model is depicted in Figure 8, and the results of the structural coverage testing are presented in Table 3.

Based on the test cases, the triggering and closing tests, as well as the performance tests at the software functional level, all meet the requirements. Special attention was paid to the structural coverage of the application layer software, with the results indicating the decision coverage of 96%, condition coverage of 100%, modified condition decision coverage of 100%, and 100% execution. The designed algorithm exhibits a high level of coverage.

### 4.2. Hardware-in-the-Loop Test

The application of algorithms in the domain controller designed in this paper primarily focuses on hazardous vehicle conditions, such as the use of RSC to prevent vehicle rollover and AYC to maintain vehicle lateral stability.

**Figure 9:**
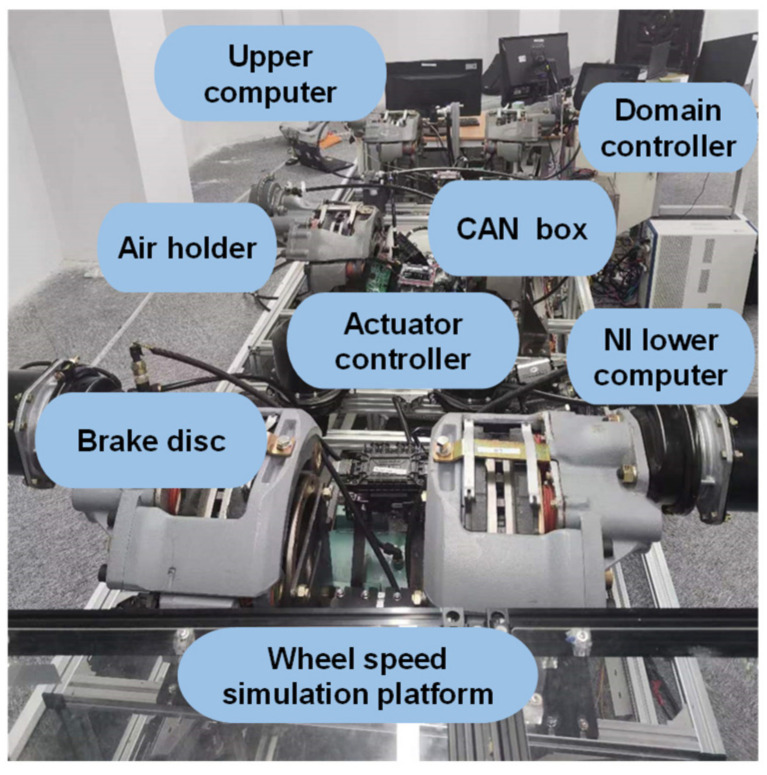
HIL test.

Through HIL testing, the effectiveness of control strategies under extreme conditions can be effectively verified, thereby avoiding potential dangers in on-road experiments. Additionally, parameter calibration of the control algorithms can be conducted through HIL simulation tests, leading to a reduction in repetitive on-road experiments and effectively shortening the development cycle and reducing the experimental costs. The HIL platform is illustrated in Figure 9, where the TruckSim vehicle model runs on the host PC, and LabVIEW-RT runs on the NI embedded system, serving as the underlying operational environment for real-time simulation. The hardware platform performs braking and transmits signals back.

**Figure 10:**
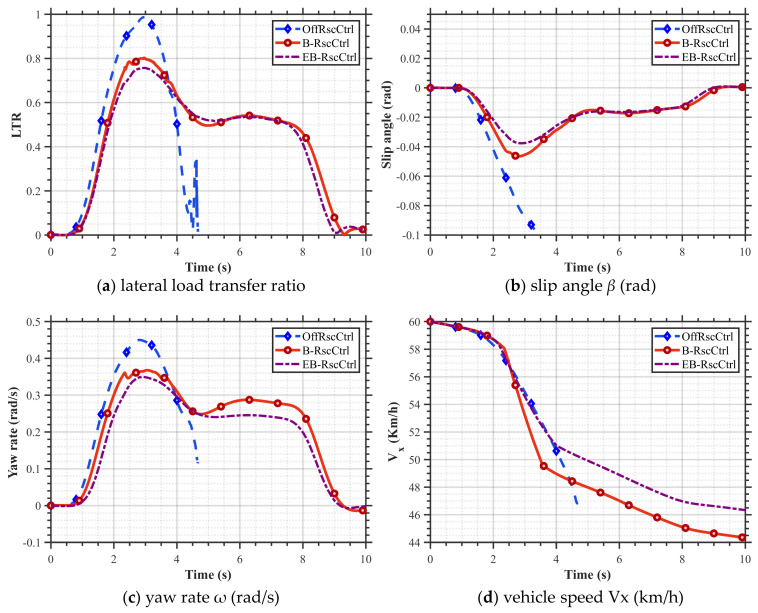
Vehicle dynamic response with different control strategies under J-turn conditions.

The experiments and tests in this article were conducted in accordance with the national standard “Performance Requirements and Test Methods for Electronic Stability Control Systems for Commercial Vehicles.” The road adhesion coefficient is set at 0.8, the initial vehicle speed is 60 km/h, and the driving path is the J-turn condition (Accelerate in a straight line, enter a circular arc with a radius of 47.6 m, and complete the J-turn condition after forming an angle of 120° in the arc). The vehicle undergoes HIL simulation under the RSC based on the combined control strategy of differential braking and engine torque limitation (EB_RscCtrl). At the same time, two strategies, namely the uncontrolled (OffRscCtrl) strategy and the conventional differential brake control (B_RscCtrl) strategy, are compared with EB_RscCtrl in this paper, and the results are shown in Figure 10.

As shown in Figure 10a and Table 4, the OffRscCtrl strategy LTR reaches 1 and experiences rollover at 3 s. The maximum LTR value for the B_RscCtrl strategy is 0.82, while the maximum LTR for the EB_RscCtrl strategy used in this paper is 0.75, representing a 9.33% reduction. The curve for the B_RscCtrl strategy exhibits oscillations between 2.3 s and 4 s due to the activation of differential braking for rollover prevention during that period, stabilizing after 9.3 s. The EB_RscCtrl strategy is observed to have a minor chance of rollover during the 2.2–2.4 s interval, prompting the activation of engine torque limitation control. During the 2.4–3.2 s interval, the risk of rollover is deemed severe, resulting in the activation of engine torque limitation control and differential brake control. Finally, during the 2.3–3.7 s interval, a slight rollover risk is detected, prompting the activation of engine torque limitation control again.

Figure 10b,c show that under OffRscCtrl conditions, the lateral deviation angle and the yaw rate rapidly diverge at around 2.5 s. However, under the B_RscCtrl and EB_RscCtrl control strategies, both *ω* and *β* eventually converge to zero. When there is a risk of rollover (2.3–4 s), the maximum *β* for the EB_RscCtrl strategy is –0.037 rad, and the maximum *ω* is 0.34 rad/s, while the maximum *β* for the differential braking control strategy is –0.046 rad, and the maximum *ω* is 0.37 rad/s. Using the EB_RscCtrl strategy reduces the maximum *β* by 19.5% and the maximum *ω* by 8.1%. In addition, it sometimes reaches steady-state in a shorter time. As shown in Figure 10d, under the EB_RscCtrl strategy, the vehicle’s speed was 46.8 km/h after 7 s of testing, which did not exceed the national standard limit of 47 km/h. After 8 s, the speed decreased to 44.7 km/h, still within the national standard limit of 45 km/h, thereby complying with the regulatory requirements.

Under the B_RscCtrl strategy, the vehicle’s speed was 46.1 km/h after 7 s of testing, which did not exceed the national standard limit of 47 km/h. After 8 s, the speed decreased to 44.3 km/h, still within the national standard limit of 45 km/h, thereby complying with the regulatory requirements. Both performance metrics meet the test requirements. However, the EB_RscCtrl strategy proposed in this paper reduces the vehicle speed to a lesser extent while still meeting the requirements and is closer to the national standard boundary. This avoids the significant reduction in vehicle speed required to meet the national standards, which would otherwise affect ride comfort. Therefore, the method in this paper improves vehicle comfort when the anti-roll control is activated.

The HIL test results show that under J-turn steering conditions, the EB_RscCtrl strategy designed in this paper can effectively prevent vehicle rollover compared to no control. Compared to the B_RscCtrl strategy, it significantly improves the vehicle’s stability and comfort under extreme conditions while effectively preventing vehicle rollover.

### 4.3. Real Vehicle Testing and Calibration

**Figure 11:**
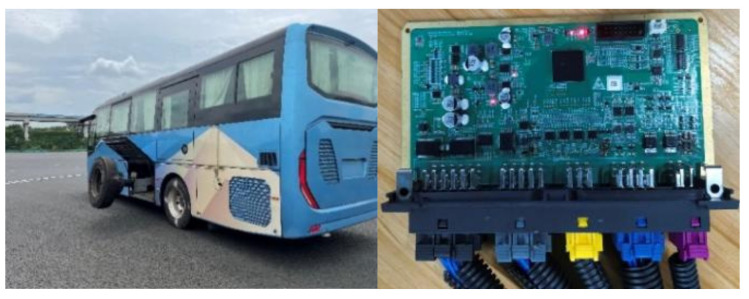
Chassis domain controller actual vehicle test environment (**left**) and hardware platform (**right**).

After conducting both MIL and HIL tests, the chassis domain controller is installed in a large coach for on-road testing at a standardized experimental test site. Due to the potential rollover risk of the tested RSC functional components, the coach needs to be equipped with rollover prevention brackets to ensure experimental safety, as shown in Figure 11. During the actual testing process, third-party software CANape is used to adjust the software calibration parameters through the XCP protocol, thereby enabling real-time parameter modification and effectively improving calibration efficiency.

In this paper, several key parameters that need to be calibrated for RSC are shown in Table 5. Among them, *judgec_pct_RSCLTRRefBra_per* is used to determine the activation of RSC. If this value is set too low, RSC will intervene too early, affecting vehicle comfort. If it is set too high, RSC will intervene too late, increasing the risk of vehicle rollover. Therefore, this value needs to be calibrated during the testing process to ensure that the RSC function meets the national standard requirements.

**Figure 12:**
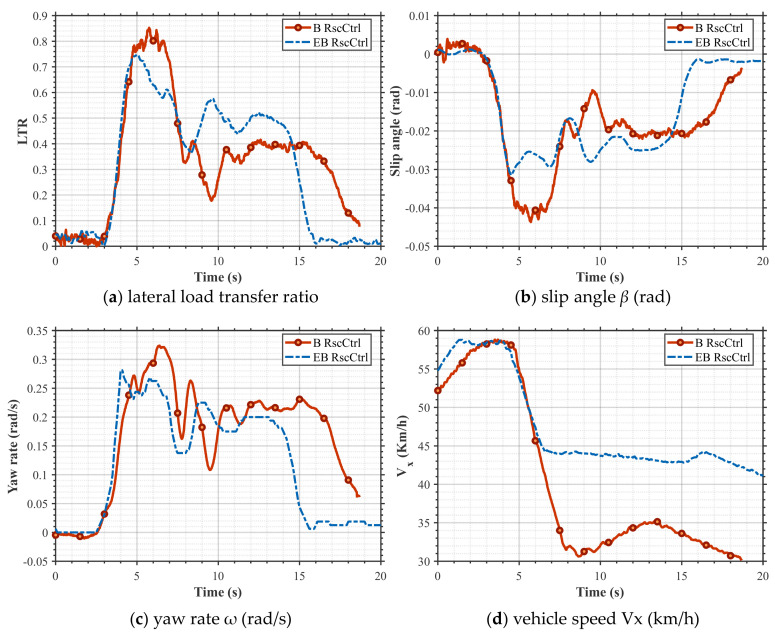
Vehicle dynamic response of J-turn control strategy under actual vehicle conditions.

Due to the risks associated with actual vehicle calibration and validation, this paper only conducts tests under EB_RscCtrl and conventional B_RscCtrl conditions and compares the final test results with the standards. The experimental road surface adhesion coefficient is 0.8, and the initial vehicle speed is 58 km/h; the results are shown in Figure 12 and Table 6. The maximum value of LTR for the B_RscCtrl strategy is 0.84, while the maximum for the EB_RscCtrl strategy used in this paper is 0.76, representing a 9.52% reduction.

Figure 12b,c show that under both the B_RscCtrl and EB_RscCtrl strategies, *ω* and *β* can ultimately converge to zero. However, in situations with a risk of rollover (4–7 s), the maximum *β* under the EB_RscCtrl strategy is −0.031 rad, and the maximum *ω* is 0.28 rad/s. In comparison, the maximum *β* under the B_RscCtrl strategy is −0.043 rad, and the maximum *ω* is 0.32 rad/s. The EB_RscCtrl strategy reduces the maximum *β* by 27.9% and the maximum *ω* by 12.5%, compared with B_RscCtrl, and reaches a steady state more quickly. As shown in Figure 12d, under the EB_RscCtrl strategy, the vehicle speed was 44.3 km/h after 7 s of testing, which did not exceed the national standard limit of 47 km/h, and the vehicle speed was 44.1 km/h after 8 s of testing, which did not exceed the national standard limit of 45 km/h, thus meeting the national standard requirements. Under the differential braking combined strategy, the vehicle’s speed was 35.1 km/h after 7 s of testing, which did not exceed the national standard limit of 47 km/h, and the vehicle speed was 31.3 km/h after 8 s of testing, which did not exceed the national standard limit of 45 km/h. The performance meets the test requirements; however, the differential braking significantly reduces the vehicle’s longitudinal speed, which affects driving comfort. The actual vehicle validation test results show that, under J-turn steering conditions, the EB_RscCtrl designed in this paper significantly improves the stability and comfort of the vehicle under extreme conditions while effectively preventing vehicle rollover, compared with the differential braking control strategy.

Overall, with the EB_RscCtrl proposed in this paper compared with the traditional B_RscCtrl, our combined torque-limiting/differential braking strategy uniquely addresses the comfort–speed trade-off. Unlike pure differential braking, which sacrifices longitudinal speed (>25% drop in our tests), EB_RscCtrl limits speed reduction to less than 10% (Figure 12d). This validates our approach’s advantage in preserving comfort without compromising safety. Meanwhile, EB_RscCtrl outperforms conventional differential braking in stability metrics (LTR, *β*, *ω*) while mitigating comfort limitations. Our approach mitigates rollover risk with smaller reductions in vehicle speed compared to conventional RSC systems, thereby enhancing ride comfort and minimizing the perception of intervention harshness. In contrast to some advanced academic strategies, the proposed system does not require any additional high-cost sensors, high-performance chips, or actuators beyond the standard configurations already present in modern braking and power systems, which ensures the feasibility of production and effective cost control.

The proposed chassis domain controller architecture is inherently scalable across different vehicle classes due to its AUTOSAR-compliant, modular software design. Each functional module, such as ABS, ASR, AYC, BM, and RSC, is implemented as an SWC with standardized interfaces, enabling seamless deployment on various ECUs regardless of hardware vendor or target platform. For adaptation to different vehicle types, including smaller passenger cars, light commercial vehicles, or autonomous vehicles, the control logic remains unchanged, while only vehicle-specific parameters—such as mass, center of gravity height, wheelbase, and braking force distribution—require recalibration. The LTR-based RSC strategy can be readily parameterized for vehicles with different dynamics, ensuring consistent rollover prevention performance.

## 5. Conclusions

This paper presents a novel approach that seamlessly integrates the AUTOSAR standard with the MBD development model, predicated on the principles of hierarchical and modular design; a chassis domain controller was designed and implemented based on a domain-centralized electronic and electrical architecture. The core tasks encompassed the software architecture design for the chassis domain controller, the development of software module components using Matlab/Simulink, and the integration testing on a domain controller hardware platform based on the Renesas u2a16 chip. This was further supplemented with HIL testing and vehicle calibration testing. The test results show that the RSC functional component in the software layer can effectively control vehicle rollover. The proposed differential brake and engine torque limitation combined control strategy keeps the center of gravity side-slip angle *β* and yaw angular velocity *ω* within a reasonable range. Compared to the uncontrolled condition, it effectively improves the vehicle’s stability under extreme conditions. Compared to conventional differential braking control, the maximum *β* is reduced by 19.5%, and the maximum *ω* is reduced by 8.1%, approaching steady state in a shorter time. The development of the chassis domain controller based on AUTOSAR achieves software–hardware decoupling and enhances the flexibility, reusability, and scalability of software development, allowing for a more focused approach to the development and iteration of functional algorithms. This improves research and development efficiency while shortening the development cycle, which is significant for the further iteration and practical application of intelligent chassis systems.

Future research will be based on AUTOSAR, and in the intelligent chassis controller, longitudinal control will be expanded to the coordinated control of longitudinal, lateral, and vertical.

## Figures and Tables

**Table 1:** RSC lower brake force distribution scheme.

Front-Wheel Angle (deg)	Trends in Rollover	Single-Side Braking Procedure
δ>0	rollover to the right	right front wheel right rear wheel
δ<0	rollover to the left	left front wheel left rear wheel

**Table 2:** The real bus parameters used in this article.

Vehicle Parameter	Value
vehicle height	3.43 m
vehicle width	2.54 m
vehicle length	8.99 m
wheelbase	4.3 m
unladen mass of vehicle	9300 kg
rolling radius of the wheel	0.451 m
front wheel track	2.09 m
rear wheel track	1.89 m
center of mass height	1.75 m

**Table 3:** RSC module structure coverage test results.

Decision	Condition	MCDC	Execution
96%	100%	100%	100%

**Table 4:** HIL test results of different control strategies under J-turn conditions.

Control Strategies	LTR Maximum	*β* Maximum/Rad	*ω* Maximum/Rad/s
OffRscCtrl	1	-	-
B_RscCtrl	0.82	−0.046	0.37
EB_RscCtrl	0.75	−0.037	0.34

**Table 5:** The core calibration parameters of the RSC template in the chassis domain controller.

Parameter Name	Value	Parameter Meanings
judgec_pct_RSCLTRRefBra_per	0.75	Reference threshold for lateral load transfer rate
rscc_fac_BraCtrlP	8	Brake control P parameter
rscc_fac_BraCtrlI	3	Brake control I parameter

**Table 6:** Actual vehicle test results of different control strategies under J-turn conditions.

Control Strategies	LTR Maximum	*β* Maximum/Rad	*ω* Maximum/Rad/s
B_RscCtrl	0.84	−0.043	0.32
EB_RscCtrl	0.76	−0.031	0.28

## Data Availability

The datasets used and/or analyzed during the current study are available from the corresponding author on reasonable request.
